# Formation and Diffusion of Metal Impurities in Perovskite Solar Cell Material CH_3_NH_3_PbI_3_: Implications on Solar Cell Degradation and Choice of Electrode

**DOI:** 10.1002/advs.201700662

**Published:** 2017-12-27

**Authors:** Wenmei Ming, Dongwen Yang, Tianshu Li, Lijun Zhang, Mao‐Hua Du

**Affiliations:** ^1^ Materials Science and Technology Division Oak Ridge National Laboratory Oak Ridge TN 37831 USA; ^2^ Key Laboratory of Automobile Materials of MOE and Department of Materials Science and Engineering Jilin University Changchun 130012 China; ^3^ State Key Laboratory of Superhard Materials Jilin University Changchun 130012 China

**Keywords:** CH_3_NH_3_PbI_3_, density functional theory, electrodes, impurities, perovskite solar cells

## Abstract

Solar cells based on methylammonium lead triiodide (MAPbI_3_) have shown remarkable progress in recent years and have demonstrated efficiencies greater than 20%. However, the long‐term stability of MAPbI_3_‐based solar cells has yet to be achieved. Besides the well‐known chemical and thermal instabilities, significant native ion migration in lead halide perovskites leads to current–voltage hysteresis and photoinduced phase segregation. Recently, it is further revealed that, despite having excellent chemical stability, the Au electrode can cause serious solar cell degradation due to Au diffusion into MAPbI_3_. In addition to Au, many other metals have been used as electrodes in MAPbI_3_ solar cells. However, how the external metal impurities introduced by electrodes affect the long‐term stability of MAPbI_3_ solar cells has rarely been studied. A comprehensive study of formation energetics and diffusion dynamics of a number of noble and transition metal impurities (Au, Ag, Cu, Cr, Mo, W, Co, Ni, Pd) in MAPbI_3_ based on first‐principles calculations is reported herein. The results uncover important general trends of impurity formation and diffusion in MAPbI_3_ and provide useful guidance for identifying the optimal metal electrodes that do not introduce electrically active impurity defects in MAPbI_3_ while having low resistivities and suitable work functions for carrier extraction.

## 1 Introduction

Recent years have witnessed enormous interests in hybrid organic–inorganic perovskite methylammonium lead iodide CH_3_NH_3_PbI_3_ (MAPbI_3_) as an exceptional solar absorber material due to the high power conversion efficiency (PCE) (>20%) in MAPbI_3_‐based solar cells and the low‐cost material synthesis using solution‐processing techniques.^1^, ^2^, ^3^, ^4^ MAPbI_3_ is an unusual solar absorber material, in which efficient carrier transport^5^, ^6^, ^7^, ^8^ coexists with a high density of defects.^9^, ^10^ This is related to the soft lattice and the large static dielectric constant (60–70)^11^, ^12^, ^13^ of MAPbI_3_, which, on one hand, promote the defect formation and, on the other hand, suppress carrier trapping at charged defects and impurities.^14^, ^15^, ^16^ The low defect formation energies in MAPbI_3_ indicate that the energy cost for bond breaking and distortion is low, which further implies low defect diffusion barriers. Indeed, fast diffusion of native defects in MAPbI_3_ 14, 17, 18, 19, 20, 21, 22, 23, 24, 25 and the resulting phenomena (such as hysteresis in current–voltage curves,^25^, ^26^, ^27^, ^28^ giant dielectric constant,^28^, ^29^ switchable photovoltaic effect,^17^ photon‐induced phase separation,^30^ etc.) have been reported and extensively discussed in the literature.

Despite extensive research on intrinsic defects in MAPbI_3_, extrinsic impurities in MAPbI_3_ are far less explored and their properties are not well understood. Impurities may have profound impact on the performance of MAPbI_3_ solar cells by introducing deep levels as nonradiative recombination centers, compensating the built‐in electric field, changing the band offset at interfaces, creating shunting paths, etc. The unintentional impurity incorporation in MAPbI_3_ could lead to significant device degradation. Many noble and transition metals (e.g., Au, Ag, Cu, Cr, Mo, Ni) have been used as electrodes in MAPbI_3_ solar cells,^31^, ^32^, ^33^, ^34^, ^35^, ^36^, ^37^, ^38^, ^39^ which are potential sources of impurity contamination for the MAPbI_3_ light absorption layer. The large dielectric constant in MAPbI_3_ should promote the formation and diffusion of not only native defects but also impurities. Thus, the metal atoms in the electrode may diffuse into the MAPbI_3_ layer as impurities and potentially cause degradation of the solar cell. Such degradation mechanism should be a serious concern for the solar cells in which the metal electrode is directly in contact with the MAPbI_3_ layer without a hole transport layer (HTL) in between. The HTL‐free MAPbI_3_ solar cells^40^, ^41^, ^42^, ^43^ have gained significant interest because the commonly used hole transport material, [2,2′,7,7′‐tetrakis(N,N‐di‐p‐methoxyphenyl‐amine) 9,9′‐spirobifluorene] (spiro‐MeOTAD), suffers from costly processing and long‐term stability issues.^44^

Even if there is a HTL separating the MAPbI_3_ layer and the metal electrode, the metal atoms may still diffuse through the HTL causing contamination of the MAPbI_3_ layer.^31^ A recent experiment based on the solar cell in the FTO/TiO_2_/MAPbI_3_/HTL/Au configuration showed that Au can diffuse into MAPbI_3_ at 70 °C, leading to significant solar cell degradation.^31^ Coating the Au electrode with a thin Cr layer provided a stable but substantially lower PCE (13% with the Cr layer vs > 20% without Cr). It is clear that the viability of MAPbI_3_‐based solar cells in the future requires the identification of electrode materials that enable a high PCE with good stability.

Zhao et al. recently reported that the MAPbI_3_ solar cell with a Cu electrode in an inverted configuration (ITO/PEDOT/MAPbI_3_/Cu) has a stable PCE (>20%) without CuI formation at the MAPbI_3_/Cu interface even after prolonged annealing of the device at 80 °C.^34^ It is puzzling why Au, a chemically more stable metal than Cu, causes significant solar cell degradation whereas Cu does not. Here, it should be cautioned that not causing solar cell degradation does not necessarily mean that metal atoms do not diffuse from the Cu electrode into MAPbI_3_ because MAPbI_3_ is known to be defect‐tolerant.^15^, ^45^, ^46^ To fully understand the effects of different metal electrodes on the performance of MAPbI_3_ solar cells, it is necessary to first understand the physical properties of metal impurities in MAPbI_3_.

In this paper, we report a comprehensive study of both energetics and kinetics of various noble and transition metal impurities in MAPbI_3_ based on first‐principles calculations. The goal is to offer comprehensive understanding of metal impurity properties in MAPbI_3_ and to identify the electrode materials that do not introduce electrically active impurity defects while having low resistivities as well as suitable work functions for carrier extraction. Specifically, we investigated the formation and the diffusion of metal impurities in MAPbI_3_ as well as their structural, electronic, and magnetic properties. The calculated formation energy of an impurity determines the impurity concentration at thermal equilibrium while the calculated impurity diffusion barrier determines the kinetic barrier for the impurity to reach its thermal equilibrium condition.

The metal impurities studied here include Au, Ag, Cu, Cr, Mo, W, Co, Ni, and Pd. All of these metals are potential electrode materials for MAPbI_3_ solar cells due to their suitable workfunctions and their relatively low resistivities (see **Table** **1**). To efficiently extract photogenerated carriers from MAPbI_3_, the Fermi level of the metal electrode should be above the valence band maximum (VBM) and below the conduction band minimum (CBM) of MAPbI_3_, which were measured to be −5.5 and −3.9 eV, respectively,^1^ Recent experimental studies show that the metal electrodes with a wide range of work functions result in similar open‐circuit voltage.^35^ The MAPbI_3_ solar cells with Au and Ag electrodes both exhibit high power conversion efficiency although their work functions are very different (Au: 5.1 eV; Ag: 4.26 eV). This may be due to the interfacial dipole formation that modifies the band alignment at the semiconductor/metal interface. Another factor affecting the choice of the metal electrode is electrical resistivity, which should be as low as possible to reduce the series resistance of the solar cell. Among the metals studied here, Au and Ag are the most widely used electrodes in MAPbI_3_ solar cells while Cu, Cr, Mo, W, and Ni electrodes have also been reported.^37^, ^38^, ^39^, ^49^, ^50^, ^51^, ^52^, ^53^ We are not aware of the use of Co or Pd electrode in MAPbI_3_ solar cells.

**Table 1:** Fermi levels relative to the vacuum energy level (in eV) and resistivities at 300 K (in × 10^−8^ Ω m) of the metals studied in this paper

	Au	Ag	Cu	Cr	Mo	W	Ni	Co	Pd
Fermi levela)	−5.1	−4.26	−4.65	−4.5	−4.6	−4.55	−5.15	−5.0	−5.12
Resistivityb)	2.271	1.629	1.725	12.7	5.52	5.44	7.20	5.6	10.80

^a)^ Ref. 47

^b)^ Ref. 48.

There are also other mechanisms that the metal electrode can influence the performance of solar cells. For example, the iodine ions in MAPbI_3_ can diffuse to the electrode surface forming iodides and causing electrode corrosion, especially when the moisture level in the environment is significant.^33^, ^34^ An electric dipole may also form at the interface between the metal electrode and the MAPbI_3_ or the charge selection layer. In addition, a large number of impurities in MAPbI_3_ may cause the modification of both the crystal structure and the electronic structure. These effects, however, are beyond the scope of this study. In this paper, we focus only on how the metal electrode affects the bulk properties of MAPbI_3_ through ion diffusion from the electrode into MAPbI_3_ and the formation of impurity defects.

## 2 Computational Approaches

Our calculations were based on density functional theory (DFT) implemented in the plane‐wave basis VASP code.^54^ The projector augmented wave method with the scalar relativistic effect was used to describe the interaction between ions and electrons.^55^ Experimental lattice constants of the room‐temperature tetragonal phase MAPbI_3_ were used: *a* = 8.849 Å and *c* = 12.642 Å.^56^, ^57^ Isolated impurities were simulated in 2 × 2 × 1 supercells. The kinetic energy cutoff of 400 eV and the 1 × 1 × 2 reciprocal space k‐point mesh were used. The atomic positions were fully relaxed until the residual forces were less than 0.02 eV Å^−1^. Extra electrons (holes) together with uniform compensating charges were added to the supercell for negatively (positively) charged impurities.

The impurity formation energy Δ*H* was calculated according to^10^

(1) $$\Delta H\;\;=\;\;(E_{D}\;\;-\;\;E_{0})\;\;-\;\;\sum_{i}n_{i}(\mu_{i}\;\;+\;\;\mu_{i}^{\mathrm{bulk}})\;\;+\;\;q(\varepsilon_{\mathrm{VBM}}\;\;+\;\;\varepsilon_{f})\;\;+\;\;\Delta E_{\mathrm{corr}}$$

Here, *E*_D_ and *E*_0_ are the total energies of the impurity‐containing and the impurity‐free supercells; *n_i_* is the difference in the number of atoms for the *i*th atomic species between the impurity‐containing and impurity‐free supercells; *μ_i_* is the chemical potential of the *i*th atomic species relative to its bulk chemical potential $\mu_{i}^{\mathrm{bulk}}$; ε_VBM_ is the energy of the VBM of the host material; ε_f_ is the Fermi energy relative to the VBM; $\Delta E_{\mathrm{corr}}^{}$ is the correction to the supercell simulation, including potential alignment and image charge corrections.^58^ The formation energy of a metal impurity was calculated assuming that the impurity is equilibrium with the electrode. Thus, the chemical potential of the impurity in MAPbI_3_ is equal to that of the bulk metal (i.e., *μ_i_* = 0 in Equation (1)). The charge transition level ε(*q*/*q*′) of an impurity was determined by the Fermi level at which the formation energy of the impurity with the charge state *q* is equal to that with the charge state *q*′

(2) $$\varepsilon (q/q\prime )=\;\;\frac{E_{D,q\prime }\;\;-\;\;E_{D,q}}{q\;\;-\;\;q\prime }$$

Optimized impurity structures, formation energies, and charge transition levels were obtained using the Perdew–Burke–Ernzerhof (PBE) exchange‐correlation functional^59^ [without the spin–orbit coupling (SOC)] while the VBM and the CBM were corrected using the more accurate Heyd–Scuseria–Ernzerhof (HSE) hybrid functional^60^, ^61^ (with 43% Fock exchange) including the SOC,^15^, ^62^ which results in a band gap of 1.50 eV, in good agreement with the experimental result of 1.51–1.52 eV.^56^, ^57^ Although the band gap obtained from the PBE calculation without SOC (1.59 eV) is also in good agreement with experiment, positions of the VBM and the CBM in the PBE calculation are both too high.^62^ Therefore, charge transition levels were calculated at the PBE level and then referenced to the VBM calculated at the HSE‐SOC level. This hybrid method has been frequently used because it has been shown previously that the band gap error in local density approximation and generalized gradient approximation does not affect the position of the defect level significantly in the absolute scale despite that the defect level position relative to the band edges is incorrect.^63^, ^64^, ^65^, ^66^, ^67^

Besides the band gap error discussed above, the PBE functional is not sufficiently accurate in describing localized electronic states (especially the transition‐metal 3d states) due to the self‐interaction error. Therefore, we further computed the impurity levels for all the interstitial impurities and selected substitutional impurities using hybrid functional HSE calculations. In HSE calculations, the structures optimized at the PBE level were adopted without further relaxation because the structural relaxation of the systems under this study by HSE calculations is extremely slow and incurs prohibitively high computational cost. A previous test on Au interstitial, ${\mathrm{Au}}_{i}^{+}$, showed that the HSE‐calculated formation energy based on the PBE‐optimized structure is in good agreement with the PBE‐calculated formation energy.^14^

HSE calculations reduce the self‐interaction error in PBE calculations; thus, resulting in the lowering of the occupied d levels. However, the large fraction of the Fock exchange α = 43% used in the exchange functional (which is necessary for reproducing the correct band gap of MAPbI_3_)^15^, ^62^ introduces too much localization for d electrons as showed by recent studies.^68^, ^69^, ^70^ Thus, the level of localization in the transition metal d states should be underestimated in PBE calculations and overestimated in HSE calculations. The true impurity levels are expected to be located between the PBE and HSE values.

DFT+*U* calculations are often used for treating transition metal d states, where *U* is the effective on‐site Coulomb interaction between d orbitals. However, we did not use the PBE+*U* method for the following reasons: (1) The empirical *U* parameter cannot be uniquely determined. The *U* parameter is typically determined by fitting to experimental results^71^, ^72^ or by self‐consistent calculations.^73^ Different values of *U* can be obtained by fitting different experimental results. For example, for binary transition metal oxides, the *U* parameters obtained by fitting to experimentally measured reaction enthalpies^71^ can differ by a few eV from those obtained by fitting to thermochemical stability trend.^72^ (2) The *U* parameter depends on the oxidation state of the transition metal ion.^73^, ^74^ This makes it difficult to calculate the charge transition levels of a transition metal impurity using the DFT+*U* method because multiple oxidation states of the transition metal ions are involved. (3) The *U* parameter depends on the chemical environment. The *U* parameters for transition metal ions were typically determined for oxides not halides in the literature. Therefore, the use of the DFT+*U* method to calculate transition levels between different oxidation states of a transition metal impurity with an open d shell has not been adequately tested and validated. In this study, we use PBE and HSE calculations to determine an energy range for each impurity level. These calculations serve two purposes: (1) determine qualitatively if the impurity introduces deep gap states; (2) determine the relevant charge states that should be considered in the diffusion barrier calculations. The results show that they serve the above purposes well. The difference in the results obtained by the PBE and the HSE calculations does not affect the conclusion of this paper. We will discuss these points in more details in Section 3.6.

The impurity defect concentration (*N*) at thermal equilibrium can be calculated by

(3) $$N\;\;=\;\;N_{0}\exp\;(-\Delta H/kT)$$

where *N*_0_ is the number of the available sites for defect formation, ΔH is the formation energy of the impurity defect, *k* is the Boltzmann constant, and *T* is temperature. The impurity diffusion barrier was calculated using the nudged elastic band method in conjunction with the climbing image method.^75^, ^76^ These calculations were performed based on the PBE functional without the SOC, which had previously been used to obtain accurate defect diffusion barriers in MAPbI_3_ and other halides.^14^, ^20^, ^77^

## 3 Results and Discussion

### 3.1 Energetic Properties and Electronic Structure of Metal Impurities

Significant diffusion of metal atoms from the electrode into the MAPbI_3_ layer requires both low formation energy and a low diffusion barrier of the metal impurity in MAPbI_3_. When a metal atom enters MAPbI_3_, it most likely diffuses through interstitial sites. Hopping through cationic MA or Pb sites requires the assistance of MA or Pb vacancies, which have high diffusion barriers.^19^, ^20^, ^21^ Thus, we focused our studies on metal impurities on interstitial sites in MAPbI_3_.

**Figure 1:**
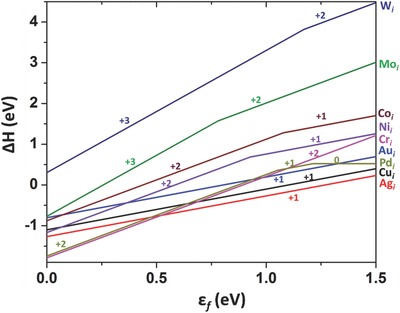
Formation energies of interstitial metal impurities in MAPbI_3_ as functions of the Fermi level. The slope of a formation energy line indicates the charge state of the impurity defect; the Fermi level at which the slope changes is the charge transition level.

The formation energy of a charged impurity in MAPbI_3_ is a function of the Fermi level (see Equation (1)), which depends on the material growth condition. The Fermi level of MAPbI_3_ is not expected to be close to the VBM or the CBM because MAPbI_3_ typically has a low free carrier concentration.^78^, ^79^ As shown in **Figure** **1**, the calculated formation energies of the interstitial metal impurities are mostly low when the Fermi level is in the midgap region, except for the cases of W*_i_* and Mo*_i_* exhibiting the formation energies above 1 eV. The highest formation energy of W*_i_* is attributed to its weak binding with I. (All metals studied here can form iodides except W.) An estimate of the impurity concentration using Equation (3) shows that, at 80 °C the thermal equilibrium concentration of an impurity is on the order of only 10^8^ cm^−3^ or less if the impurity formation energy is >1 eV. Thus, W and Mo electrodes should have negligible effects on the bulk properties of semi‐insulating MAPbI_3_.

**Figure 2:**
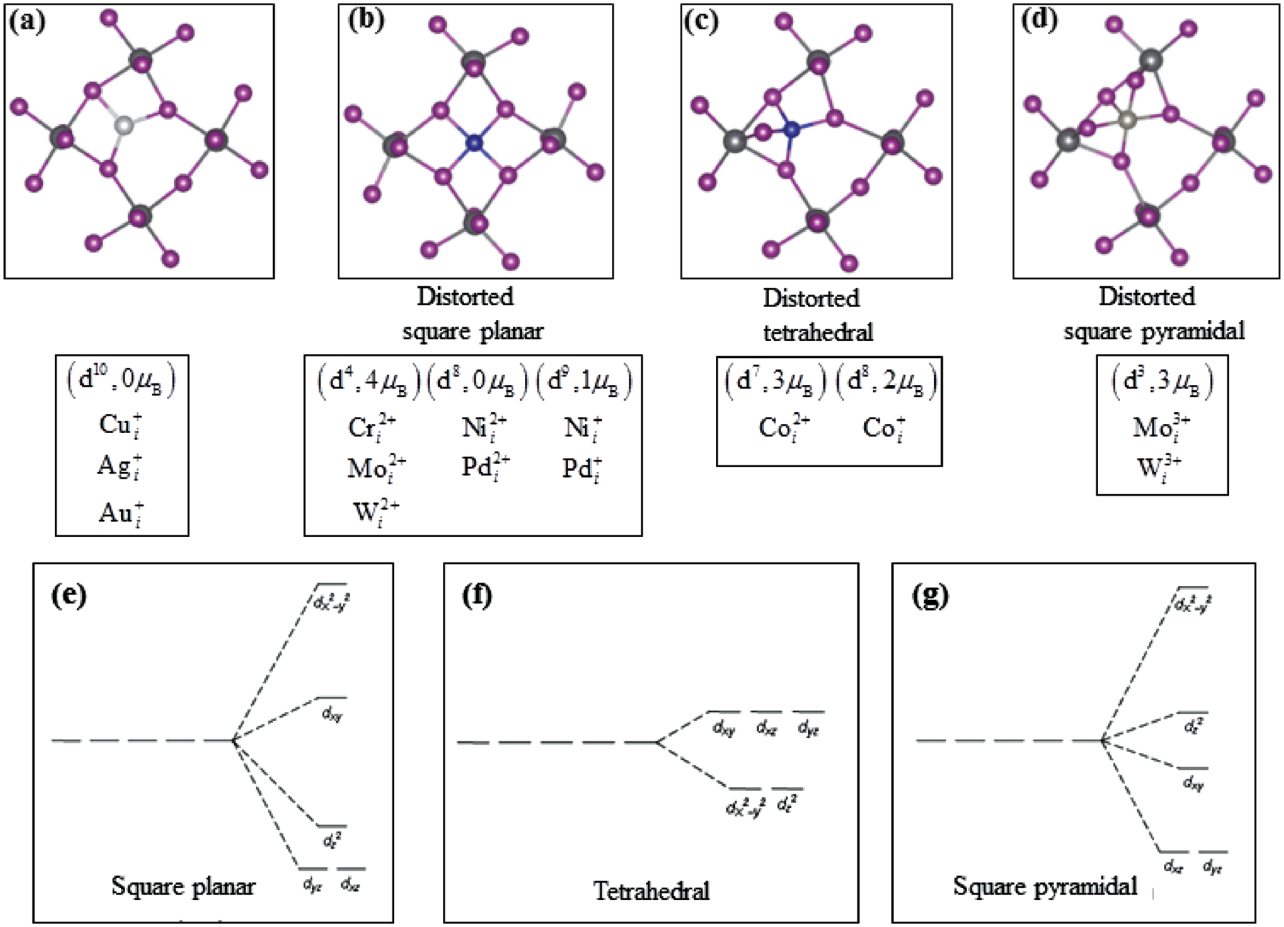
Four structural motifs for interstitial metal impurities in MAPbI_3_: a) three metal–iodine bonds: ${\mathrm{Cu}}_{i}^{+}$, ${\mathrm{Ag}}_{i}^{+}$, and ${\mathrm{Au}}_{i}^{+}$; b) four metal–iodine bonds in a distorted square planar structure: ${\mathrm{Cr}}_{i}^{2+}$, ${\mathrm{Mo}}_{i}^{2+}$, $W_{i}^{2+}$, ${\mathrm{Ni}}_{i}^{2+}$, ${\mathrm{Pd}}_{i}^{2+}$, ${\mathrm{Ni}}_{i}^{+}$, and ${\mathrm{Pd}}_{i}^{+}$; c) four metal–iodine bonds in a distorted tetrahedral structure: ${\mathrm{Co}}_{i}^{2+}$, ${\mathrm{Co}}_{i}^{+}$; d) five metal–iodine bonds in a distorted square pyramidal structure: ${\mathrm{Mo}}_{i}^{3+}$ and $W_{i}^{3+}$; and schematic diagrams for crystal field splitting of d levels in e) square planar, f) tetrahedral, and g) square pyramidal structures.

The ground‐state structures of the interstitial metal impurities (summarized in **Figure** **2**) are complicated and are determined by multiple factors, such as the charge state, the crystal field, and the magnetic moment. However, there is a general trend displayed in Figure 2, that is, an interstitial metal impurity binds with iodine ions and its coordination number tends to increase with the charge state of the impurity. The monovalent [${\mathrm{Cu}}_{i}^{+}$, ${\mathrm{Ag}}_{i}^{+}$, and ${\mathrm{Au}}_{i}^{+}$ (Figure 2a)], the divalent [${\mathrm{Cr}}_{i}^{2+}$, ${\mathrm{Mo}}_{i}^{2+}$,$W_{i}^{2+}$, ${\mathrm{Ni}}_{i}^{2+}$, ${\mathrm{Pd}}_{i}^{2+}$, and ${\mathrm{Co}}_{i}^{2+}$ (Figure 2b,c)], and the trivalent [${\mathrm{Mo}}_{i}^{3+}$and $W_{i}^{3+}$ (Figure 2d)] metal impurities bind with three, four, and five I^−^ neighbors, respectively. Such trend can be understood by the stronger Coulomb attraction between a higher‐valent metal impurity and I^−^ ions, which further leads to the trend that higher charge state of an impurity generally corresponds to higher diffusion barrier as discussed in Section 3.3.

The divalent interstitial impurities shown above all take a distorted square planar structure [Figure 2b] except ${\mathrm{Co}}_{i}^{2+}$. The square planar crystal field splits the d‐orbital energy levels as shown in Figure 2e. ${\mathrm{Cr}}_{i}^{2+}$, ${\mathrm{Mo}}_{i}^{2+}$, and $W_{i}^{2+}$ are all d^4^ ions and take the high‐spin state (4 μ_B_). ${\mathrm{Ni}}_{i}^{2+}$ and ${\mathrm{Pd}}_{i}^{2+}$ are d^8^ ions and take the low‐spin state (0 μ_B_) because taking the high‐spin state (2 μ_B_) would require the filling of the spin‐up $d_{x^{2}-\;y^{2}}$ level, which is high in energy and energetically unfavorable to occupy. ${\mathrm{Co}}_{i}^{2+}$ is a d^7^ ion and takes the high‐spin state (3 μ_B_) in a distorted tetrahedral structure (Figure 2c) because the tetrahedral crystal field is relatively weak, which favors the high‐spin state.

${\mathrm{Co}}_{i}^{+}$ is a d^8^ ion and takes a high‐spin state (2 μ_B_) in a distorted tetrahedral structure unlike the other d^8^ ions (${\mathrm{Ni}}_{i}^{2+}$ and ${\mathrm{Pd}}_{i}^{2+}$) that take a low‐spin state of 0 μ_B_ in a distorted square planar structure. This is because, in contrast to ${\mathrm{Ni}}_{i}^{2+}$ and ${\mathrm{Pd}}_{i}^{2+}$, ${\mathrm{Co}}_{i}^{+}$ has a lower charge state, consequently longer metal–iodine bonds. The long bonds and the distorted tetrahedral structure create a relatively weak crystal field, which promotes the high‐spin state. ${\mathrm{Mo}}_{i}^{3+}$ and $W_{i}^{3+}$ are d^3^ ions and take a high‐spin state (3 μ_B_) in distorted square pyramidal structures (Figure 2d,g). ${\mathrm{Pd}}_{i}^{0}$, which may be stabilized in n‐type MAPbI_3_ (see Figure 1), is in d^10^ configuration and has no magnetic moment. Since it is charge neutral, its ground‐state structure (Figure S1d, Supporting Information) is coordinated with one Pb^2+^ and three I^−^ on the *ab*‐plane to enhance charge polarization and Coulomb binding.

### 3.2 Diffusion of Interstitial Metal Impurities

**Figure 3:**
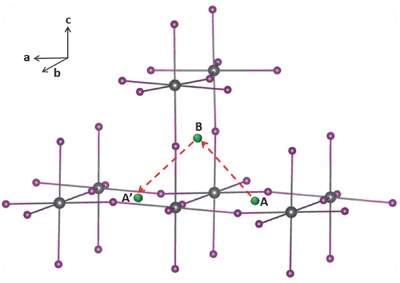
Schematic of the diffusion path of an interstitial metal impurity in MAPbI_3_.

**Figure 4:**
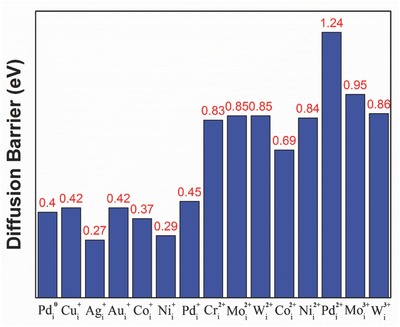
Diffusion barriers (in eV) of interstitial metal impurities at different charge states in MAPbI_3_. The diffusion path is from the point A to the point B in Figure 3. If there are multiple barriers along the diffusion path, the highest barrier is shown.

Having low formation energy does not necessarily mean that the metal impurity would have a high concentration in MAPbI_3_ because the kinetic barrier may obstruct the diffusion of the metal impurity from the electrode into MAPbI_3_. We next calculated diffusion barriers for all interstitial metal impurities at all possible charge states shown in Figure 1. The identified energetically favorable diffusion path is schematically depicted in **Figure** **3**, in which the impurity hops from the interstitial site A on the *ab*‐plane to the interstitial site B on the *bc*‐plane followed by another equivalent hop to another interstitial A′ site on the *ab*‐plane. The diffusion barriers were calculated by taking the site A as the starting point and the site B as the final point, and the obtained values are shown in **Figure** **4**.

**Figure 5:**
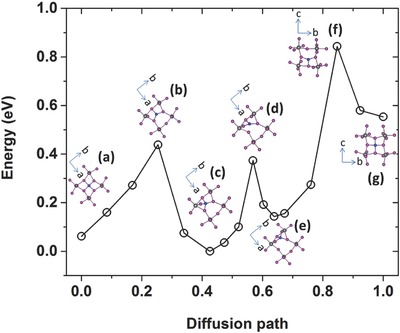
Potential energy evolution along the diffusion path of ${\mathrm{Co}}_{i}^{2+}$. Insets show the a) initial and f) final structures as well as the structures of the c,e) metastable and b,d) transition states along the diffusion path. The insets in (a)–(e) are viewed from the [001] direction and the insets in (f) and (g) are viewed from the [010] direction.

The lattice of MAPbI_3_ is very plastic, which is evidenced by many potential wells on the potential energy landscape. We found for several impurities that multiple metastable sites exist along the diffusion path, which results in multiple kinetic barriers (see **Figure** **5** for an example). This may enhance the impurity diffusion rate because, instead of hopping over a high barrier, the impurity can go through several lower barriers (as discussed below). Along the multibarrier diffusion path, the rate‐limiting process is the hopping over the highest barrier, which is shown in Figure 4.

The results in Figure 4 reveal a general trend that the metal impurity at a higher charge state tends to have a higher diffusion barrier. This is related to the results shown in Figure 2 that a higher charge state generally leads to a higher coordination number of the metal impurity, thereby, increasing the number of bonds that may need to be broken for the impurity to diffuse. This general trend on the metal impurity diffusion in MAPbI_3_ is expected to hold in other halide perovskites because the underlying mechanism that gives rise to this trend is electrostatic Coulomb interaction, which is dominant in halide perovskites.

The monovalent ${\mathrm{Cu}}_{i}^{+}$, ${\mathrm{Ag}}_{i}^{+}$, and ${\mathrm{Au}}_{i}^{+}$ have very low diffusion barriers (0.27–0.42 eV) in MAPbI_3_. These diffusion barriers are comparable to that of the iodine vacancy,^14^, ^20^ which is known experimentally to diffuse efficiently in MAPbI_3_.^18^ The low formation energies (Figure 1) and the low diffusion barriers (Figure 4) of ${\mathrm{Cu}}_{i}^{+}$, ${\mathrm{Ag}}_{i}^{+}$, and ${\mathrm{Au}}_{i}^{+}$ indicate that Cu, Ag, and Au can diffuse into MAPbI_3_ from their respective electrodes at room temperature.

Most of the interstitial transition metal impurities in MAPbI_3_ can have multiple charge states depending on the Fermi level (see Figure 1): Mo*_i_* and W*_i_* have the +2 and +3 charge states; Co*_i_* and Ni*_i_* have the +2 and +1 charge states; Pd*_i_* has the +2, +1, and neutral charge states; only Cr*_i_* is stable at just one charge state of +2. ${\mathrm{Cr}}_{i}^{2+}$, ${\mathrm{Mo}}_{i}^{2+/3+}$, and $W_{i}^{2+/3+}$ have sufficiently high diffusion barriers (above 0.8 eV) to render them largely immobile at room temperature. Co*_i_*, Ni*_i_*, and Pd*_i_* are also largely immobile (at the +2 charge state) at room temperature unless in n‐type samples, in which Co*_i_*, Ni*_i_*, and Pd*_i_* take lower charge states (Figure 1) and exhibit lower diffusion barriers.

While metal impurities in the d^10^ configuration (closed d shell) all diffuse from the point A to the point B in Figure 3 by hopping over a single kinetic barrier (see Figure S1, Supporting Information), the diffusion of transition metal impurities with partially filled d levels (open d shell), however, involves multiple barriers. This is because the different crystal field splitting of the d levels along the diffusion path strongly affects the energy of the transition metal impurity with an open d shell but has little effects on those with a closed d shell. Figure 5 shows the diffusion path of ${\mathrm{Co}}_{i}^{2+}$ as an example. We chose the starting point of the diffusion on the *ab*‐plane with ${\mathrm{Co}}_{i}^{2+}$ binding with four equatorial I^−^ in a square planar structure (point A in Figure 3). At the final point, ${\mathrm{Co}}_{i}^{2+}$ is bonded with two equatorial I^−^ from the two adjacent PbI_2_ layers and two apical I^−^ (point B in Figure 3).

Due to the relatively high ionicity in MAPbI_3_, the metal diffusion path largely involves breaking and making of metal–I bonds; the bonding between the metal impurity and Pb ions was not found in the diffusion path. The entire process of Co diffusion requires the breaking of three Co—I bonds and the making of three new Co—I bonds. This process is completed in three steps, each of which overcomes a relatively small barrier (0.39, 0.40, and 0.69 eV) involving the breaking of only one bond and the making of a new one. The starting and the final states as well as the two metastable states along the diffusion path all have four Co—I bonds, whereas all the transition states (saddle points) have three Co—I bonds (see insets of Figure 5 for their structures). The rate‐limiting barrier is the highest one of 0.69 eV, which is significantly smaller than the difference between the lowest minimum and the highest maximum of the potential energy along the entire diffusion path (0.84 eV). Therefore, involving intermediate steps in the diffusion path reduces the effective barrier of impurity diffusion. Other transition metal impurities with open d shell also take multibarrier diffusion paths (Figures S2–S5, Supporting Information); however, the metastable sites along their diffusion paths can be different from that of ${\mathrm{Co}}_{i}^{2+}$. For example, Cr*_i_*, Mo*_i_*, and W*_i_* pass through local energy minima corresponding to the structures with five metal–iodine bonds similar to that shown in Figure 2d.

### 3.3 Impurity‐Induced Deep Levels in MAPbI_3_

**Figure 6:**
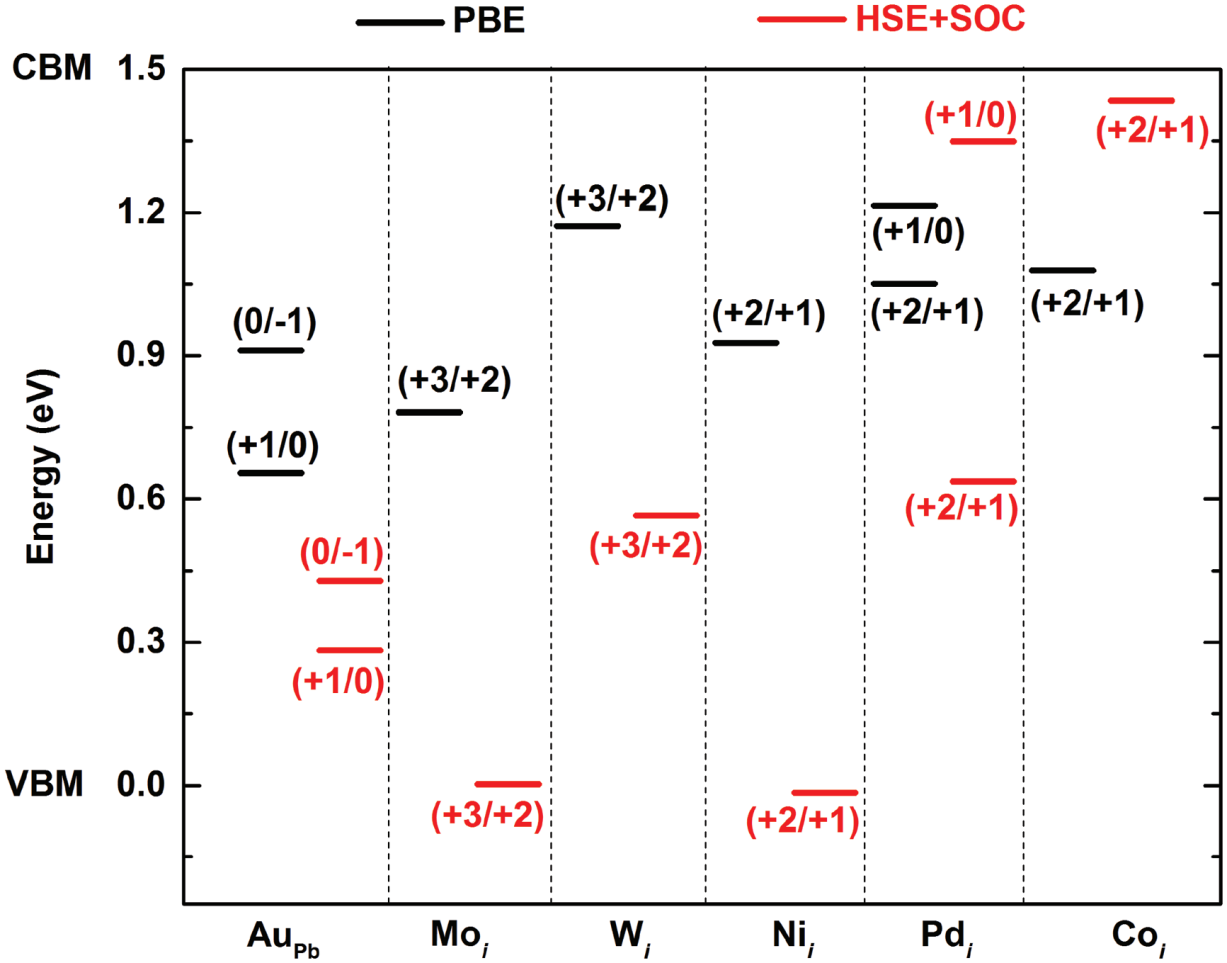
Charge transition levels for Au_Pb_, Mo*_i_*, W*_i_*, Ni*_i_*, Pd*_i_*, and Co*_i_* calculated using both the PBE functional (without the SOC) and the HSE functional (including the SOC).

Since the formation energies and the diffusion barriers of ${\mathrm{Cu}}_{i}^{+}$, ${\mathrm{Ag}}_{i}^{+}$, and ${\mathrm{Au}}_{i}^{+}$ are both low (see Figures 1 and 4), Cu, Ag, and Au ions may diffuse from their respective electrode into the MAPbI_3_ layer and affect the performance of MAPbI_3_ solar cells. Both PBE and HSE calculations show that ${\mathrm{Cu}}_{i}^{+}$, ${\mathrm{Ag}}_{i}^{+}$, and ${\mathrm{Au}}_{i}^{+}$ do not introduce deep levels inside the band gap of MAPbI_3_; thus, they do not affect carrier transport significantly. However, due to the abundance of the MA and Pb vacancies in MAPbI_3_,^9^, ^10^ ${\mathrm{Cu}}_{i}^{+}$, ${\mathrm{Ag}}_{i}^{+}$, and ${\mathrm{Au}}_{i}^{+}$ may be easily trapped by vacancies to form substitutional impurities. PBE calculations show that, while trapping of ${\mathrm{Cu}}_{i}^{+}$, ${\mathrm{Ag}}_{i}^{+}$, ${\mathrm{Au}}_{i}^{+}$ by $V_{\mathrm{MA}}^{-}$ (forming ${\mathrm{Cu}}_{\mathrm{MA}}^{0}$, ${\mathrm{Ag}}_{\mathrm{MA}}^{0}$, ${\mathrm{Au}}_{\mathrm{MA}}^{0}$) changes the total energy by 0.08, −0.27, and −0.19 eV, respectively, trapping by $V_{\mathrm{Pb}}^{2-}$ (forming ${\mathrm{Cu}}_{\mathrm{Pb}}^{-}$, ${\mathrm{Ag}}_{\mathrm{Pb}}^{-}$, ${\mathrm{Au}}_{\mathrm{Pb}}^{-}$) is energetically more favorable with the energy change of −0.50, −0.63, and −0.31 eV. Further calculations show that these substitutional impurities induce only shallow levels inside the band gap of MAPbI_3_ except ${\mathrm{Au}}_{\mathrm{Pb}}^{}$ (calculated at both PBE and HSE levels; see **Figure** **6**). The deep level of ${\mathrm{Au}}_{\mathrm{Pb}}^{}$ is due to the presence of Au‐5d levels inside the band gap. In contrast, the Cu‐3d and the Ag‐4d levels are resonant with the valence band. The higher Au‐5d levels in MAPbI_3_ are likely due to the stronger crystal field splitting at ${\mathrm{Au}}_{\mathrm{Pb}}^{}$. [Although the Cu‐3d levels are usually higher in energy than the Au‐5d levels, at ${\mathrm{Cu}}_{\mathrm{Pb}}^{}$, the small‐sized Cu is displaced away from the center of the octahedron, resulting in only threefold coordination with iodine ions, consequently, a weak crystal field (see Figure S6, Supporting Information)]. ${\mathrm{Au}}_{\mathrm{Pb}}^{}$ can trap both electrons and holes and the trapping levels are very deep, which should render ${\mathrm{Au}}_{\mathrm{Pb}}^{}$ a highly efficient nonradiative recombination center. This may explain the observed significant solar cell degradation upon Au diffusion from the Au electrode into MAPbI_3_.^31^ On the contrary, the performance of MAPbI_3_ solar cells with Cu electrodes was found to be stable even after prolonged annealing at elevated temperatures.^34^ This may be due to the absence of Cu‐induced deep levels. The Cu impurities introduced by the diffusion from the Cu electrode may behave as benign defects like intrinsic vacancies in MAPbI_3_, which do not cause serious carrier trapping despite having significant concentrations.^62^

Besides ${\mathrm{Au}}_{i}^{}$, ${\mathrm{Ag}}_{i}^{}$, and Cu_*i*_, we also calculated charge transition levels of other interstitial impurities shown in Figure 1. Both PBE and HSE calculations show that ${\mathrm{Cr}}_{i}^{}$ does not introduce deep levels inside the band gap of MAPbI_3_. The positions of gap states introduced by Mo_*i*_, W_*i*_, Ni_*i*_, Pd_*i*_, and ${\mathrm{Co}}_{i}^{}$ calculated by both PBE and HSE methods are shown in Figure 6. As discussed in Section 3, the level of localization in the transition metal d states should be underestimated in PBE calculations and overestimated in HSE calculations; the true impurity levels are expected to be located between the PBE and HSE values. The results in Figure 6 suggest that ${\mathrm{Mo}}_{i}^{}$, $W_{i}^{}$, ${\mathrm{Ni}}_{i}^{}$, ${\mathrm{Pd}}_{i}^{}$, and ${\mathrm{Co}}_{i}^{}$ are deep centers in MAPbI_3_ and, thus, should have detrimental effects on carrier transport.

As shown in Figure 4, ${\mathrm{Pd}}_{i}^{}$, ${\mathrm{Co}}_{i}^{}$, and ${\mathrm{Ni}}_{i}^{}$ at low charge states (+1 and 0) have low diffusion barriers. The diffusion of ${\mathrm{Pd}}_{i}^{}$, ${\mathrm{Co}}_{i}^{}$, and ${\mathrm{Ni}}_{i}^{}$, which are likely efficient carrier traps due to their deep levels, should be suppressed by lowering the Fermi level to promote the high charge state (+2), which corresponds to higher diffusion barriers as shown in Figure 4.

### 3.4 Implications on the Choice of Electrodes

Au, Ag, and Cu have the lowest resistivities among all the metals studied here. The low formation energies and the low diffusion barriers for ${\mathrm{Cu}}_{i}^{+}$, ${\mathrm{Ag}}_{i}^{+}$, and ${\mathrm{Au}}_{i}^{+}$ suggest that Cu, Ag, and Au can diffuse into MAPbI_3_ when they are used as electrodes; however, only Au occupying the Pb site can induce deep levels inside the band gap, resulting in efficient nonradiative carrier recombination and solar cell degradation. This result explains the experimentally observed Au‐diffusion‐induced solar cell degradation.^31^ Thus, when Au is the electrode, care should be taken to prevent Au diffusion into MAPbI_3_. For example, a dense pin‐hole‐free HTL should be used in the solar cell to separate the Au electrode and the MAPbI_3_ layer. Cu and Ag can also diffuse in MAPbI_3_ but their impurity defects are electrically benign and are much less detrimental than Au to carrier transport in MAPbI_3_. This result is consistent with the high PCE achieved in MAPbI_3_ solar cells with Cu electrodes (ITO/PEDOT/MAPbI_3_/Cu).^34^ Note that the formation of metal iodides on the surface of the electrode can also cause solar cell degradation.^33^ However, this degradation mechanism is beyond the scope of this work, which focuses on the effects of metal impurities on the properties of bulk MAPbI_3_.

${\mathrm{Cr}}_{i}^{2+}$ has low formation energy but a high diffusion barrier; thus, its diffusion into MAPbI_3_ may be kinetically hindered. It was found experimentally that inserting a thin Cr layer between the Au electrode and the HTM prevents the Au diffusion into MAPbI_3_ and leads to a lower but stable PCE,^31^ which suggests that Cr diffusion into MAPbI_3_ is likely insignificant. However, Cr has the highest resistivity among all the metals investigated here and should increase the series resistance of the solar cell if used as the electrode.

Mo*_i_* and W*_i_* have high diffusion barriers in MAPbI_3_. Furthermore, unlike other metal impurities studied here, Mo*_i_* and W*_i_* are energetically unfavorable to form in semi‐insulating MAPbI_3_. Thus, Mo and W electrodes should be most stable against metal impurity diffusion into MAPbI_3_. The MAPbI_3_ solar cells with Mo and W electrodes have been reported to exhibit satisfactory PCE (11–15%).^37^, ^53^ In particular, a study on Mo‐electrode‐based MAPbI_3_ solar cells showed a high PCE of 15.06%, very small current‐voltage hysteresis, and a mechanically durable Mo electrode.^37^

Ni*_i_*, Pd*_i_*, and Co*_i_* have moderate formation energies; their diffusion barriers are high at the +2 charge state (favored in p‐type MAPbI_3_) but low at lower charge states. Thus, p‐type MAPbI_3_ should be used to couple the Ni, Pd, or Co electrodes. Ni, Co, and Pd have work functions close to that of Au and their Fermi levels are slightly above the VBM and below the Fermi level of MAPbI_3_; thus, they may be very effective in hole extraction from MAPbI_3_ or HTM through Ohmic contact. The Ni electrode has been used in MAPbI_3_ solar cells^38^, ^39^, ^53^ whereas the Co and Pd electrodes have not been reported according to the best of our knowledge. The PCEs of 12.18^53^ and 10.4%^38^ have been reported for the solar cells with the common TiO_2_/MAPbI_3_/HTM/Ni device architecture. The use of transition metal electrodes (such as Mo, W, Ni) in MAPbI_3_ solar cells is expected to lead to somewhat reduced PCE (likely due to the higher resistivity) compared to the noble metal (Au and Ag) electrodes, but may offer improved stability and reduced cost.

### 3.5 PBE versus HSE Calculations

As discussed in Section 3, the level of localization in the transition metal d states should be underestimated in PBE calculations and overestimated in HSE calculations; the true impurity levels are expected to be located between the PBE and HSE values. With this in mind, the results in Figure 6 indicate that the differences between PBE and HSE calculations do not affect main conclusions in this study. The interstitial impurities in Figure 6 introduce deep levels inside the band gap of MAPbI_3_ and thus are harmful to the performance of MAPbI_3_ solar cells. The HSE calculations lower the (3+/2+) transition levels of Mo*_i_* and W*_i_* (relative to those from the PBE calculations); but the conclusion that these two impurities do not diffuse into MAPbI_3_ is not affected because the diffusion barriers of Mo*_i_* and W*_i_* are high at both the +3 and the +2 charge states (Figure 4). Compared to the PBE results, the (2+/+) transition levels of Pd*_i_* and Co*_i_* obtained from the HSE calculations are also changed but remain high enough to stabilize ${\mathrm{Pd}}_{i}^{2+}$ and ${\mathrm{Co}}_{i}^{2+}$ (which have high diffusion barriers) in p‐type MAPbI_3_. The (2+/+) level of Ni*_i_* calculated using the HSE functional is near the VBM. However, as mentioned above, the true (2+/+) level should be somewhere between the PBE and HSE results inside the band gap. Thus, the main conclusion regarding Ni, Pd, and Co electrodes in MAPbI_3_ solar cells remains valid, that is, p‐type MAPbI_3_ should be used to stabilize the higher charge state of the impurity, which corresponds to a relatively high diffusion barrier that prevents metal ion diffusion from the electrode to MAPbI_3_.

### 3.6 Role of ns^2^ Ions in Formation and Diffusion of Impurities

Finally, we comment that the high diffusivity of defects and impurities in MAPbI_3_ as discussed above has also been observed in other halides such as TlBr. MAPbI_3_ and TlBr both have ns^2^ ions (Pb^2+^ in MAPbI_3_ and Tl^+^ in TlBr), which have the outermost electron configuration of ns^2^. The ns^2^ ions are responsible for the large Born effective charges and the resulting large static dielectric constant in these materials, which promotes the formation and the diffusion of defects and impurities.^14^, ^15^, ^67^, ^77^, ^80^, ^81^ It has been reported that Cu, Ag, and Au can diffuse through the TlBr single crystal from one electrode to the other whereas Cr cannot [the device structure is Au/Cr/TlBr/(Cu,Ag,Au)],^82^ similar to the behavior of Cu, Ag, Au, and Cr diffusion in MAPbI_3_ as reported here.

## 4 Concluding Remarks

DFT calculations were performed to systematically study structures, magnetic properties, formation energies, and diffusion barriers of Cu, Ag, Au, Cr, Mo, W, Ni, Pd, and Co impurities in MAPbI_3_. We focused on the potential role of the metal impurities in the degradation of the MAPbI_3_ solar cell when these metals are used as back contacts. We find that, when the Fermi level is near the midgap of MAPbI_3_, the formation energies of the interstitial metal impurities are low except for Mo*_i_* and W*_i_*. All metal impurities studied here introduce detrimental deep levels in the band gap of MAPbI_3_ except Cu and Ag. The diffusion barriers of the interstitial metal impurities tend to increase with the charge state of the impurity. ${\mathrm{Cu}}_{i}^{+}$, ${\mathrm{Ag}}_{i}^{+}$, ${\mathrm{Au}}_{i}^{+}$, ${\mathrm{Co}}_{i}^{+}$, ${\mathrm{Ni}}_{i}^{+}$, and ${\mathrm{Pd}}_{i}^{+}$ have low diffusion barriers and can diffuse in MAPbI_3_ while ${\mathrm{Cr}}_{i}^{+}$, ${\mathrm{Mo}}_{i}^{2+/3+}$, $W_{i}^{2+/3+}$, ${\mathrm{Co}}_{i}^{2+}$, ${\mathrm{Ni}}_{i}^{2+}$, and ${\mathrm{Pd}}_{i}^{2+}$ have much higher diffusion barriers, rendering them largely immobile at room temperature. These results show that the choice of the electrode can have profound impact on the performance and the stability of a MAPbI_3_ solar cell. The general trend revealed by our calculations, that is, the interstitial metal impurity with a higher charge state in MAPbI_3_ tends to have a higher diffusion barrier, can serve as a simple guidance for choosing the electrode based on its potential for causing contamination and degradation in the solar cell. Cu and Ag electrodes may cause metal impurity diffusion into MAPbI_3_ but they do not introduce deep levels. When Au is the electrode, care must be taken to prevent Au diffusion into MAPbI_3_ since Au_Pb_ can cause efficient nonradiative recombination. Mo and W electrodes should be most stable against metal impurity diffusion into MAPbI_3_ due to the high formation energies and the high diffusion barriers of W*_i_* and Mo*_i_*. ${\mathrm{Cr}}_{i}^{2+}$ has low formation energy but a high diffusion barrier; thus, its diffusion into MAPbI_3_ from the Cr electrode may be kinetically hindered. p‐type MAPbI_3_ should be used to couple the Ni, Pd, and Co electrodes in order to suppress the formation of ${\mathrm{Co}}_{i}^{+}$, ${\mathrm{Ni}}_{i}^{+}$, and ${\mathrm{Pd}}_{i}^{+}$, which have low diffusion barriers, and promote the formation of ${\mathrm{Co}}_{i}^{2+}$, ${\mathrm{Ni}}_{i}^{2+}$, and ${\mathrm{Pd}}_{i}^{2+}$, which have higher diffusion barrier. These factors concerning the stability of the solar cell should be considered together with the resistivity and the work function of the metal electrode to determine the optimal electrode for MAPbI_3_ solar cells.

## Conflict of Interest

The authors declare no conflict of interest.

## Supporting information

Supplementary
