# Supplementary material for: Formation and Diffusion of Metal Impurities in Perovskite Solar Cell Material CH3NH3PbI3: Implications on Solar Cell Degradation and Choice of Electrode
Source: Adv Sci (Weinh). 2017 Dec 27;5(2):1700662. doi: 10.1002/advs.201700662 (PMC5827569; doi:10.1002/advs.201700662)
Supplement: Supplementary file 1 — Supplementary [file ADVS-5-1700662-s001.pdf]

Copyright WILEY-VCH Verlag GmbH & Co. KGaA, 69469 Weinheim, Germany, 2017.

ADVANCED SCIENCE Open Access

Supporting Information

for *Adv. Sci.,* DOI: 10.1002/advs.201700662

Formation and Diffusion of Metal Impurities in Perovskite Solar Cell Material $CH_3NH_3PbI_3$: Implications on Solar Cell Degradation and Choice of Electrode

Wenmei Ming, Dongwen Yang, Tianshu Li, Lijun Zhang, and Mao-Hua Du**

Supporting Information

Formation and diffusion of metal impurities in perovskite solar cell material $CH_3NH_3PbI_3$: implications on solar cell degradation and choice of electrode

Wenmei Ming[1,†], Dongwen Yang[2, †], Tianshu Li,[2] Lijun Zhang[2,3], and Mao-Hua Du[1]

[1]Materials Science and Technology Division, Oak Ridge National Laboratory, Oak Ridge, TN 37831, USA

[2]Key Laboratory of Automobile Materials of MOE and Department of Materials Science and Engineering, Jilin University, Changchun 130012, China

[3]State Key Laboratory of Superhard Materials, Jilin University, Changchun 130012, China

[†]Both authors contributed equally to this work.

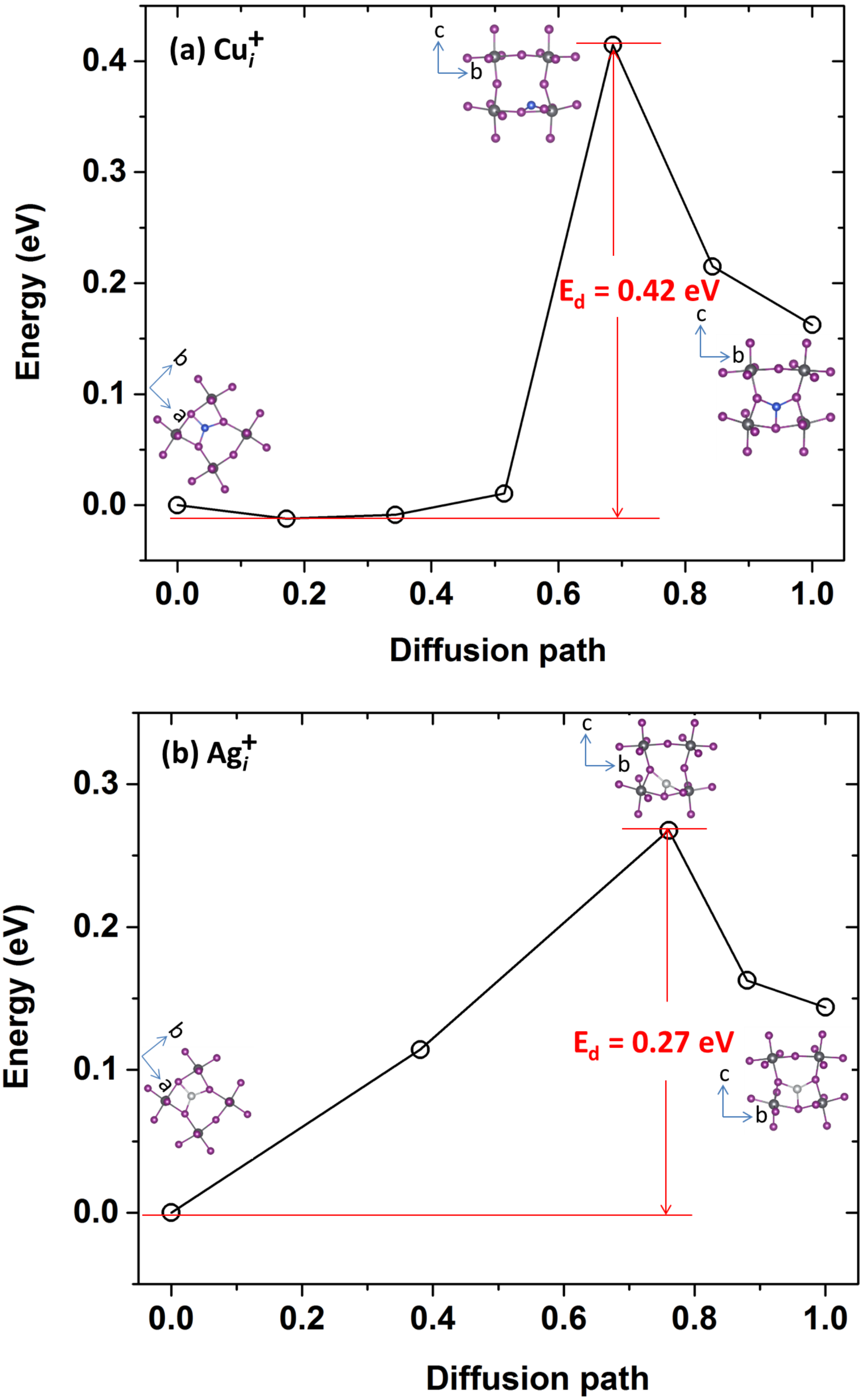

(a) Cu$_i^+$
Energy (eV)
Diffusion path
E_d = 0.42 eV
0.0
0.1
0.2
0.3
0.4
0.0
0.2
0.4
0.6
0.8
1.0
a
b
c
(b) Ag$_i^+$
Energy (eV)
Diffusion path
E_d = 0.27 eV
0.0
0.1
0.2
0.3
0.0
0.2
0.4
0.6
0.8
1.0

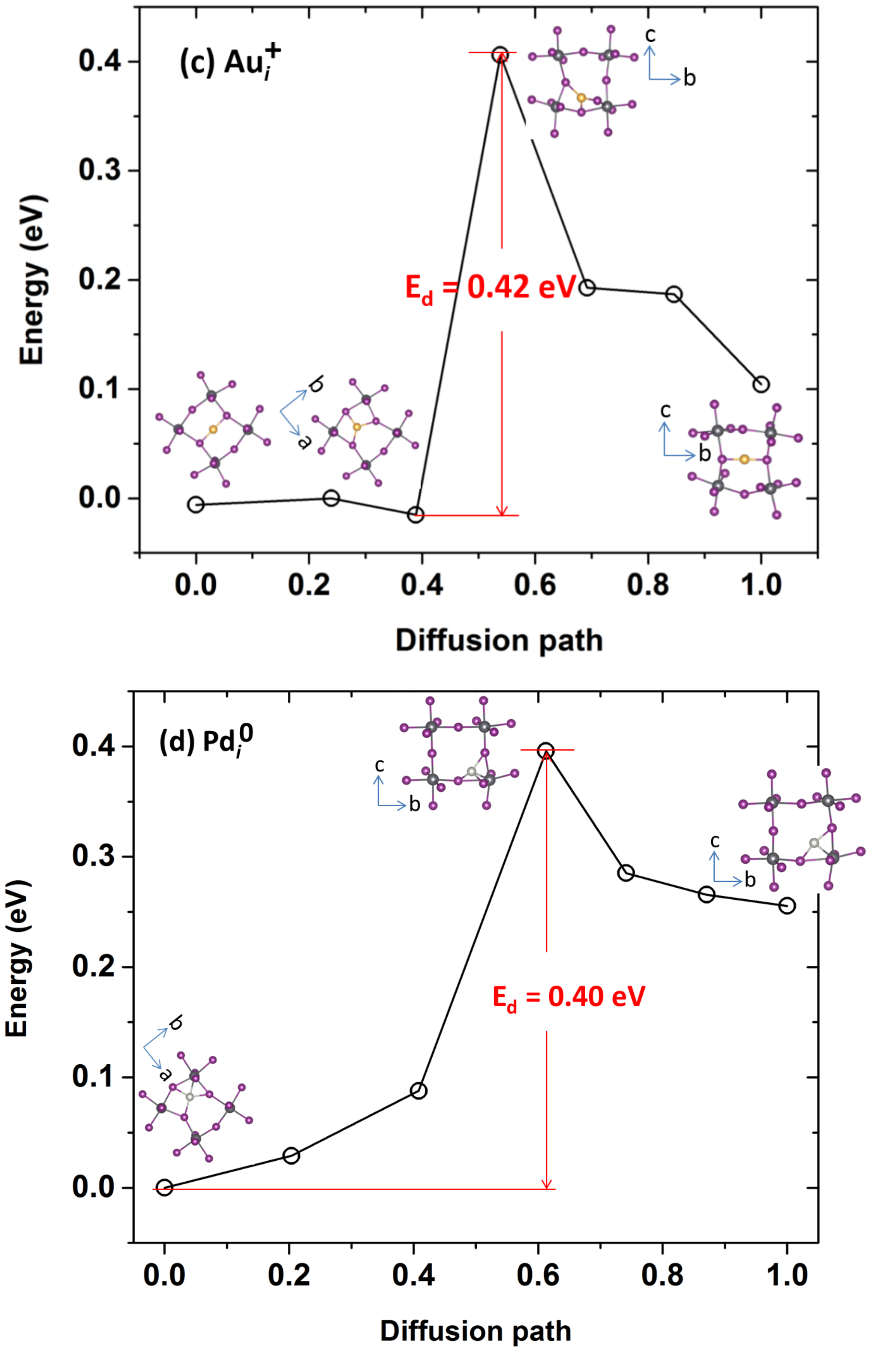

Figure S1. Diffusion path and rate limiting barrier E_d of (a) Cu_i^+, (b) Ag_i^+, (c) Au_i^+ and (d) Pd_i^0. The local structures around the impurity at its metastable sites and transition state sites along the diffusion path are given in the inset. The corresponding orientations of the structures are provided by the coordinate axis labeled with each case.

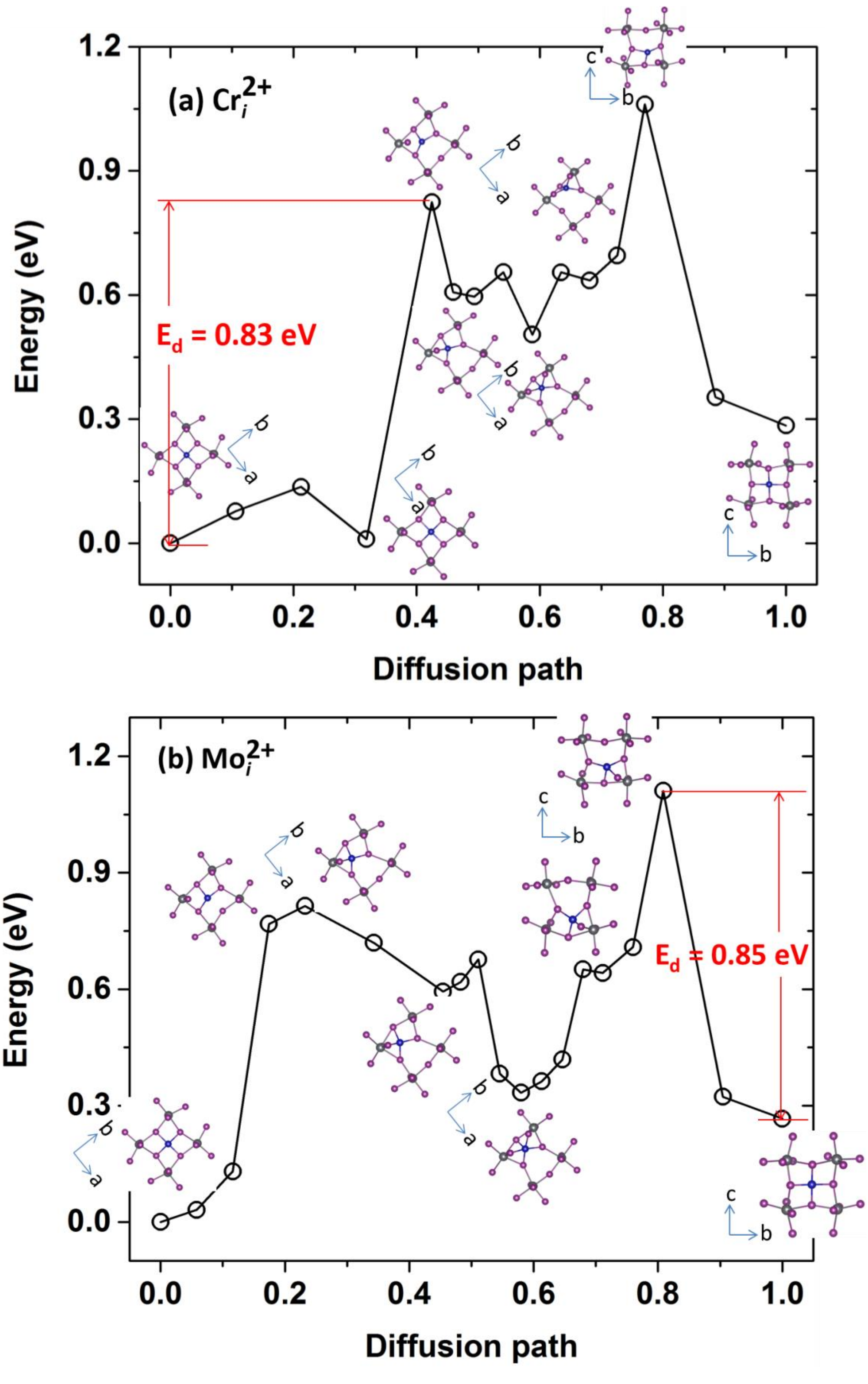

(a) Cr_i^{2+}
E_d = 0.83 eV
Energy (eV)
Diffusion path
(b) Mo_i^{2+}
E_d = 0.85 eV
Energy (eV)
Diffusion path

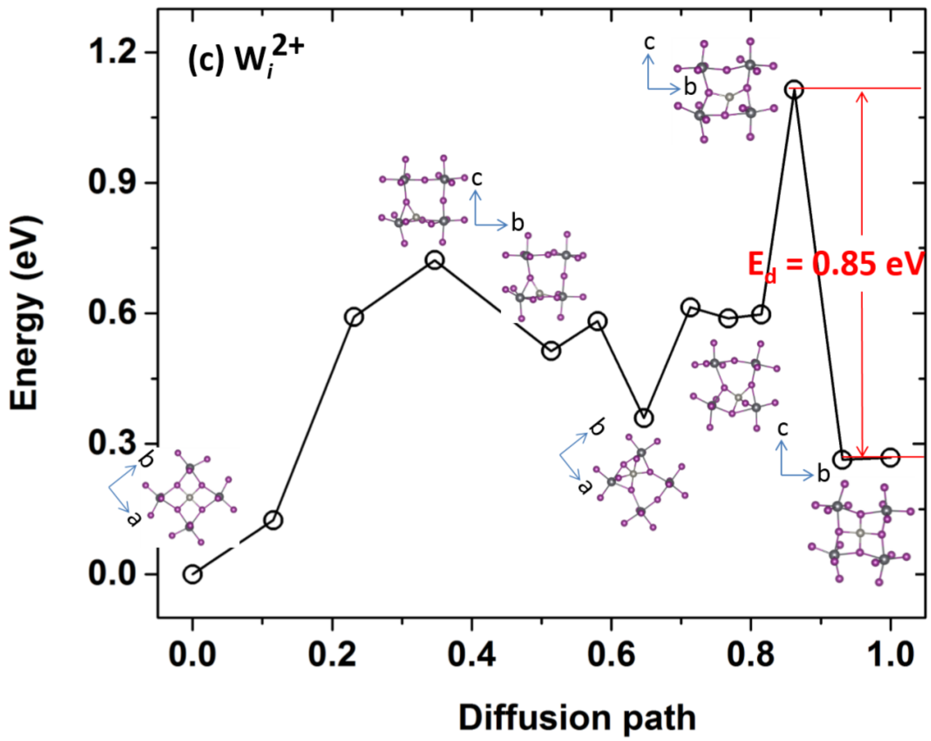

Figure S2. Diffusion path and rate limiting barrier E_d of (a) Cr_i^{2+}, (b) Mo_i^{2+}, and (c) W_i^{2+}. The local structures around the impurity at its metastable sites and transition state sites along the diffusion path are given in the inset. The corresponding orientations of the structures are provided by the coordinate axis labeled with each case.

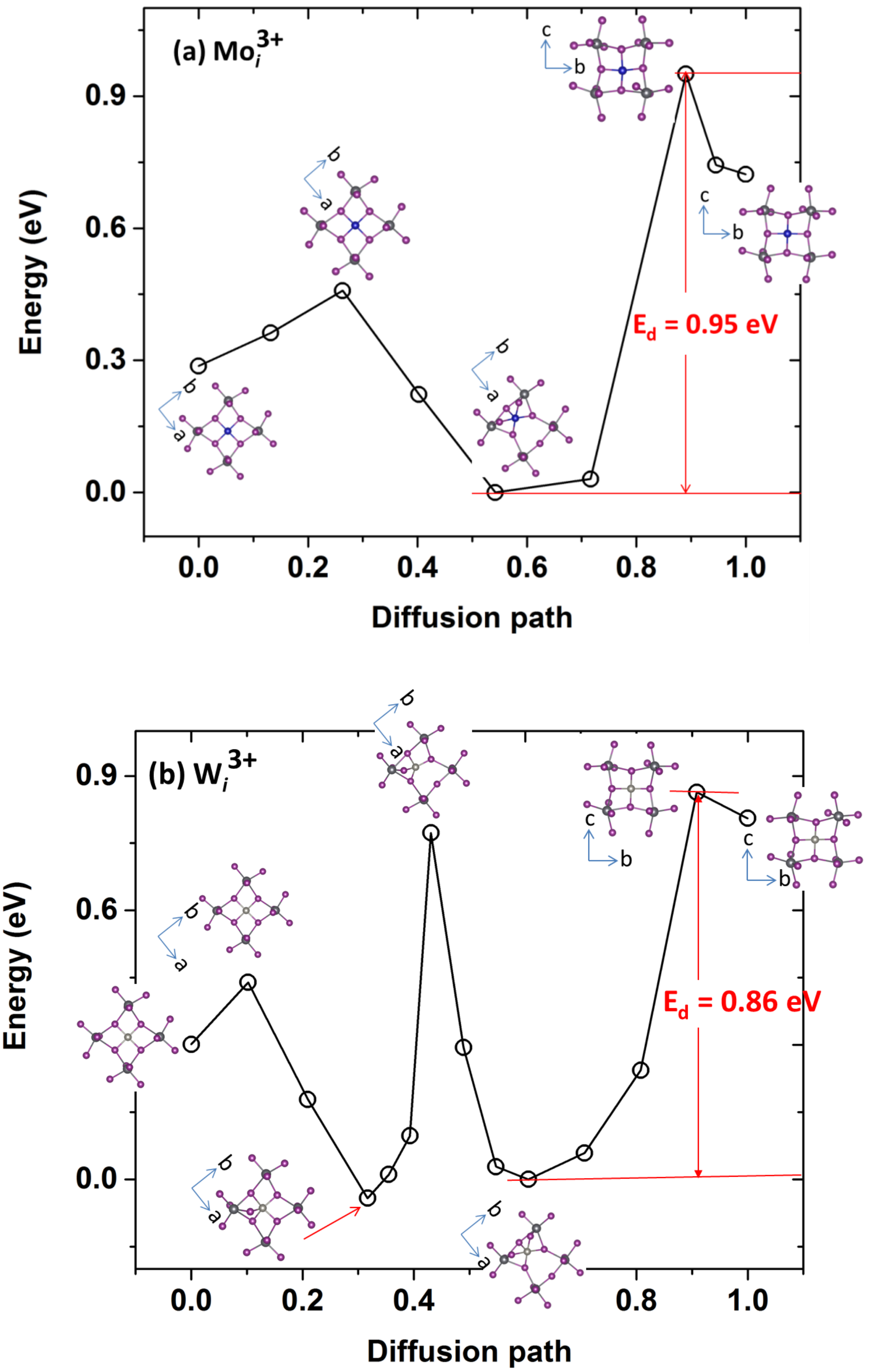

Figure S3. Diffusion path and rate limiting barrier E_d of (a) Mo_i^{3+} and (b) W_i^{3+}. The local structures around the impurity at its metastable sites and transition state sites along the diffusion path are given in the inset. The corresponding orientations of the structures are provided by the coordinate axis labeled with each case.

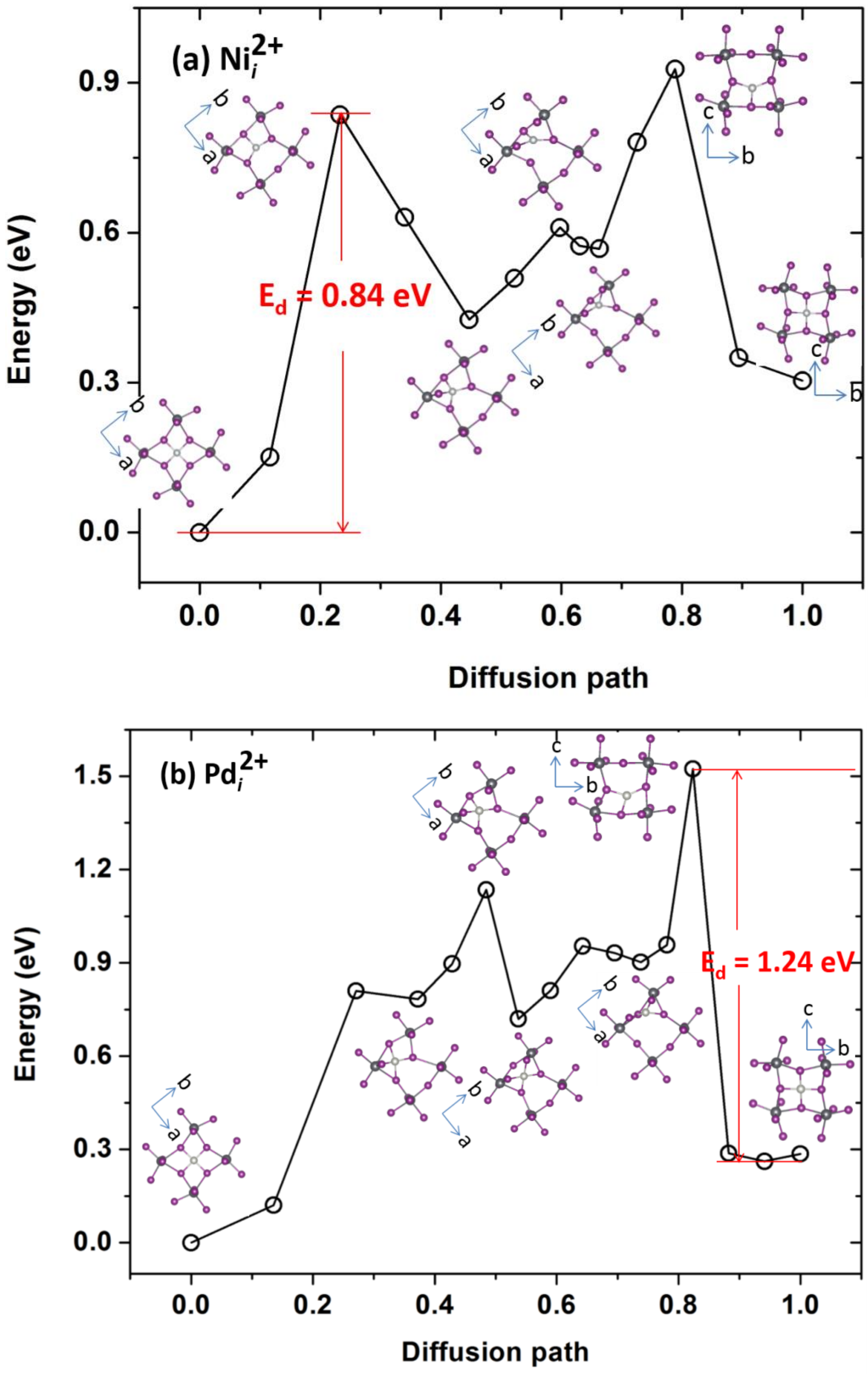

Figure S4. Diffusion path and rate limiting barrier E_d of (a) Ni_i^{2+} and (b) Pd_i^{2+}. The local structures around the impurity at its metastable sites and transition state sites along the diffusion path are given in the inset. The corresponding orientations of the structures are provided by the coordinate axis labeled with each case.

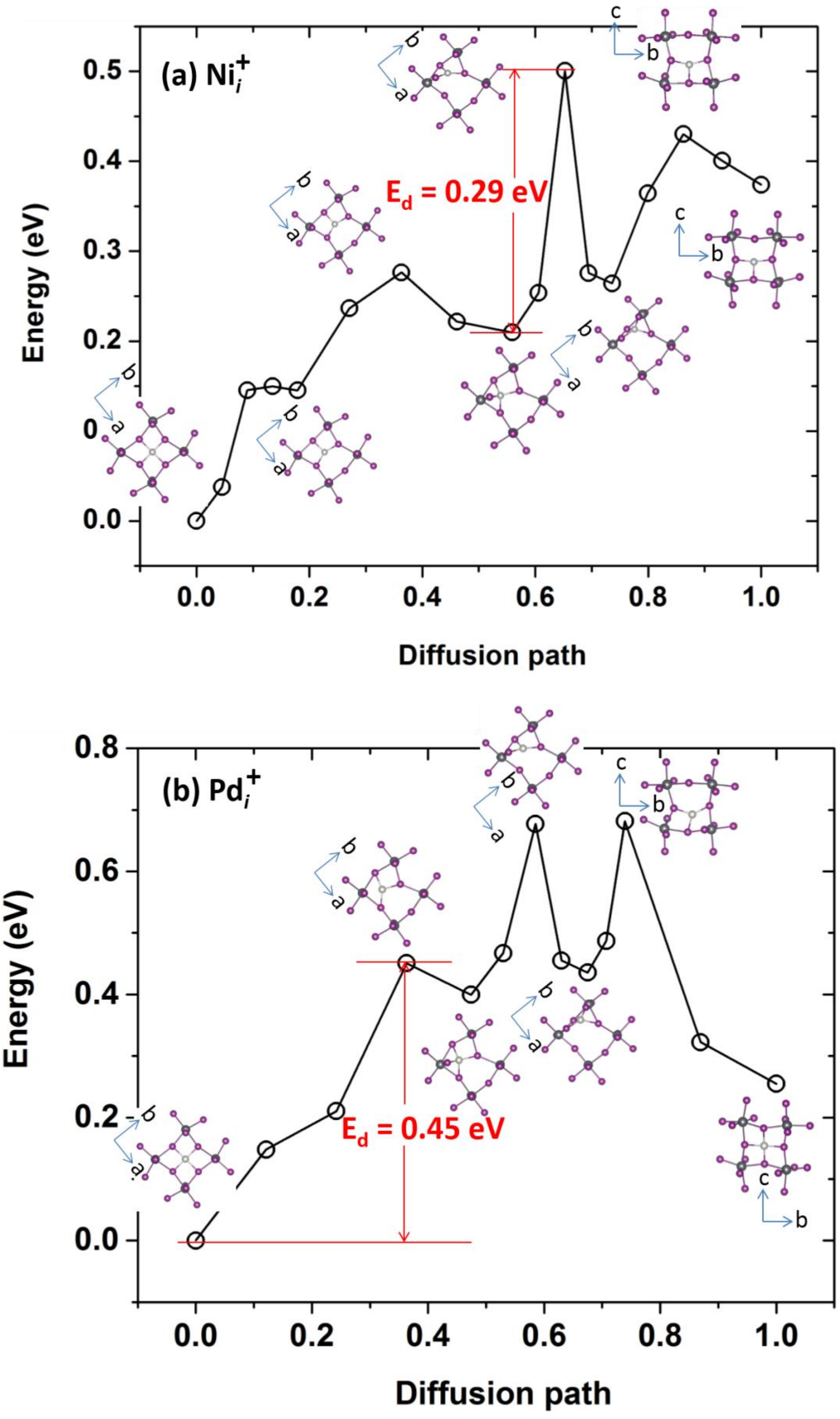

(a) Ni_i^+
E_d = 0.29 eV
Energy (eV)
0.0
0.1
0.2
0.3
0.4
0.5
Diffusion path
0.0
0.2
0.4
0.6
0.8
1.0
a
b
c
(b) Pd_i^+
E_d = 0.45 eV
Energy (eV)
0.0
0.2
0.4
0.6
0.8
Diffusion path
0.0
0.2
0.4
0.6
0.8
1.0

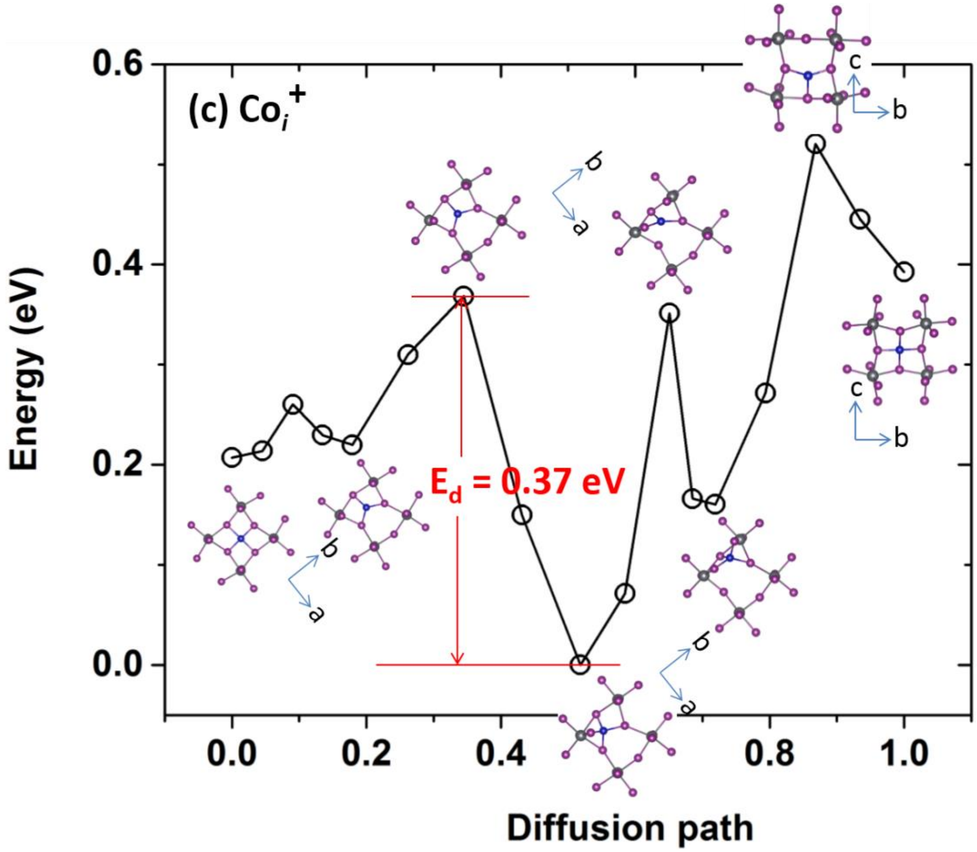

Figure S5. Diffusion path and rate limiting barrier E_d of (a) Ni_i^+, (b) Pd_i^+, and (c) Co_i^+. The local structures around the impurity at its metastable sites and transition state sites along the diffusion path are given in the inset. The corresponding orientations of the structures are provided by the coordinate axis labeled with each case.

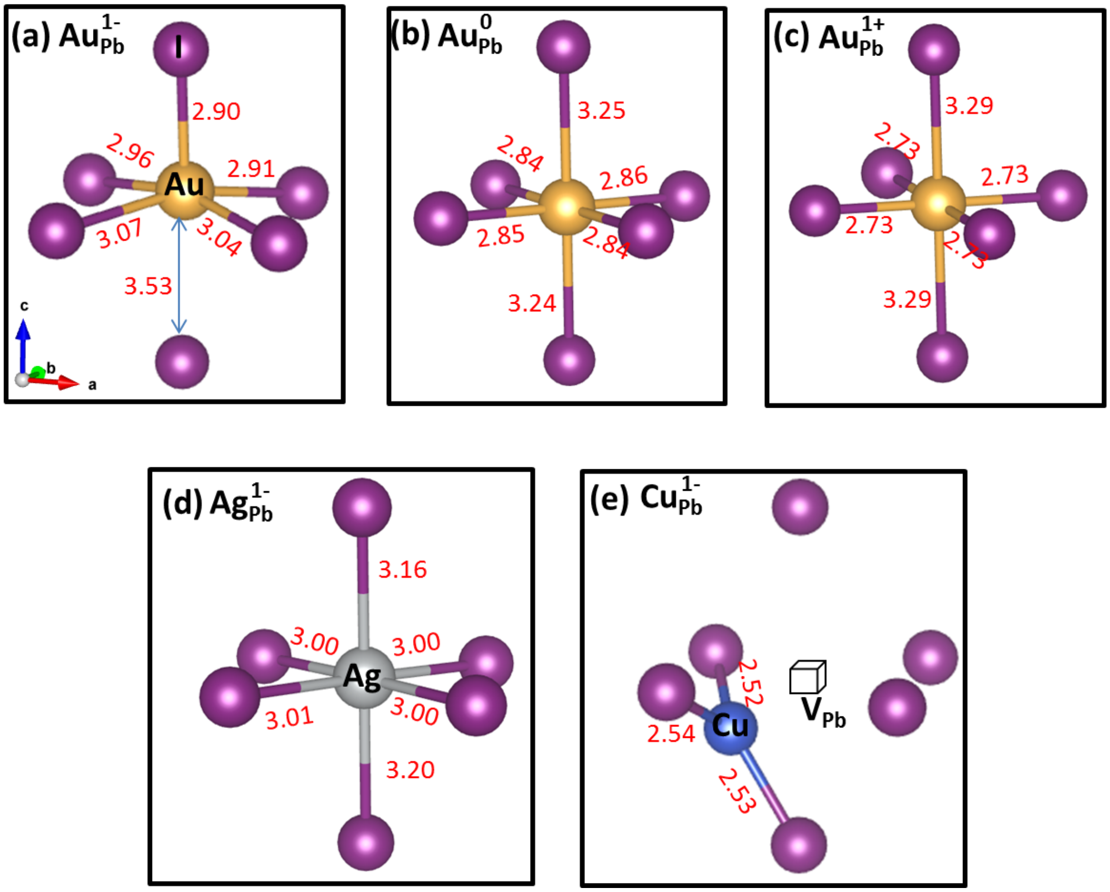

Figure S6. Structures of (a) Au_{Pb}^{-}, (b) Au_{Pb}^{0}, (c) Au_{Pb}^{+}, (d) Ag_{Pb}^{-}, and Cu_{Pb}^{-}. The numbers (in Å) give the interatomic distances.
